# The Combined Effect of Individual and Neighborhood Socioeconomic Status on Nasopharyngeal Cancer Survival

**DOI:** 10.1371/journal.pone.0073889

**Published:** 2013-09-12

**Authors:** Ting-Shou Chang, Chun-Ming Chang, Ta-Wen Hsu, Yaoh-Shiang Lin, Ning-Sheng Lai, Yu-Chieh Su, Kuang-Yung Huang, Hung-Lung Lin, Ching-Chih Lee

**Affiliations:** 1 Department of Otolaryngology, Kaohsiung Veterans General Hospital, Kaohsiung, Taiwan; 2 Department of Surgery, Buddhist Dalin Tzu Chi General Hospital, Chiayi, Taiwan; 3 Department of Otolaryngology, Buddhist Dalin Tzu Chi General Hospital, Chiayi, Taiwan; 4 Department of Internal Medicine, Buddhist Dalin Tzu Chi General Hospital, Chiayi, Taiwan; 5 Center for Clinical Epidemiology and Biostatistics, Buddhist Dalin Tzu Chi General Hospital, Chiayi, Taiwan; 6 Cancer Center, Buddhist Dalin Tzu Chi General Hospital, Chiayi, Taiwan; 7 Department of Research, Buddhist Dalin Tzu Chi General Hospital, Chiayi, Taiwan; 8 Division of Rheumatology, Department of Internal Medicine, Buddhist Dalin Tzu Chi General Hospital, Chiayi, Taiwan; 9 School of Medicine, Tzu Chi University, Hualian, Taiwan; 10 Community Medicine Research Center and the Institute of Public Health, National Yang-Ming University, Taipei, Taiwan; 11 Department of Education, Buddhist Dalin Tzu Chi General Hospital, Chiayi, Taiwan; University of Campinas, Brazil

## Abstract

**Background:**

The relationship between individual and neighborhood socioeconomic status (SES) and mortality rates in patients with nasopharyngeal carcinoma (NPC) is unknown. This population-based study aimed to examine the association between SES and survival of patients with NPC in Taiwan.

**Materials and Methods:**

A population-based follow-up study was conducted of 4691 patients diagnosed with NPC between 2002 and 2006. Each patient was traced to death or for 5 years. Individual SES was defined by enrollee job category. Neighborhood SES was based on household income dichotomized into advantaged and disadvantaged areas. Cox proportional hazards model was used to compare the death-free survival rates between the different SES groups after adjusting for possible confounding factors and risk factors.

**Results:**

In NPC patients below the age of 65 years, 5-year overall survival rates were worst for those with low individual SES living in disadvantaged neighborhoods. After adjusting for patient characteristics (age, gender, Charlson Comorbidity Index Score), NPC patients with low individual SES residing in disadvantaged neighborhoods were found to have a 2-fold higher risk of mortality than patients with high individual SES residing in advantaged neighborhoods. We found no significant difference in mortality rates between different SES groups in NPC patients aged 65 and above.

**Conclusions:**

Our findings indicate that NPC patients with low individual SES who live in disadvantaged neighborhoods have the higher risk of mortality than their more privileged counterparts. Public health strategies and welfare policies would be well advised to try to offset the inequalities in health care and pay more attention to addressing the needs of this vulnerable group.

## Introduction

Nasopharyngeal carcinoma (NPC) is common in Asia, especially southern China. The annual incidence in western countries is <1 per 100,000 population, whereas in Taiwan it is 6.17 per 100,000 [1]. Because of the difficulty treating nasopharyngeal tumors surgically, this disease is usually treated by concurrent radio- and chemotherapy or radiotherapy alone [2]. NPC is the second most common cancer in incidence and mortality (after oral cavity cancer) among the head and neck cancers in Taiwan.

Several clinical and pathologic factors have been consistently shown to influence survival in NPC patients. One social parameter, socioeconomic status (SES), has been linked to survival in several common cancers, including breast, prostate and lung [3]–[5]. In the United States, head and neck cancer patients who are not well-insured or who come from low SES neighborhoods have also been found to have poorer outcomes [6], [7]. However, there is scant information about SES-mortality relationship in NPC. In addition, most SES studies have been limited by a single, crude measure of SES, ignoring the multidimensional concept of this construct which incorporates such factors as household and neighborhood [8]. Van Jaarsveld et al. suggest that individual deprivation and neighborhood deprivation can affect health through partly different pathways [9]. A number of previous studies have linked neighborhood SES to morbidity, mortality, and engagement in health risk behaviors among residents [3], [10]–[13], Studying oropharyngeal cancer patients in a single, large multidisciplinary cancer center in Texas, Reitzel et al. found an association between a high level of neighborhood deprivation and poorer overall survival, independent of patient-level sociodemograhpic and clinical variables, including individual-level annual household income [14]. However, no study has investigated the relationship between nasopharyngeal cancer, which is highly prevalent in south Asia, and these SES factors. In this study, we merged population-based claims data obtained from Taiwan’s National Health Insurance Research Database (NHIRD) with neighborhood SES information to investigate the contextual effect of individual and neighborhood SES on NPC survival rates in Taiwan.

## Materials and Methods

### Ethics Statement

This study was approved by the Institutional Review Board of Buddhist Dalin Tzu Chi General Hospital, Taiwan. Review board requirements for written informed consent were waived because all personal identifying information was removed from the dataset prior to analysis.

### Database

The data for this study were collected from Taiwan’s NHIRD for the years 2002 to 2006. This dataset is organized and managed by Taiwan’s National Health Research Institutes but collected by Taiwan’s National Health Insurance Program, which has been in place in Taiwan since 1995. The program covers approximately 99% of the residents in Taiwan and has contracts with 97% of the medical providers there [15]. To verify accuracy of diagnosis, Taiwan’s Bureau of National Health Insurance randomly reviews the charts of one per 100 ambulatory and one per 20 inpatient claims and interviews patients [16], [17]. Due to the protection of personal confidential data, cancer stage and dietary habits could not be linked to primary survey data and were not included in this dataset.

Our study cohort consisted of Taiwan’s incidental nasopharyngeal cancer patients (*International Classification of Diseases, Ninth Revision, Clinical Modification* [ICD-9-CM] codes 147.9) who began either radiotherapy, chemotherapy, or chemoradiotherapy for their disease between 2002 and 2006.

### Measurement

The key dependent variable of interest was 5-year overall survival rate, not cause-specific survival rate because it was not possible to determine cause-specific survival rates based on the registry data we used. The use of overall survival data should not interfere significantly with our results because, as Roohan et al. have shown in a study adapting a clinical morbidity index for use with ICD-9-CM administrative databases, there is no significant difference between survival models for all-cause-mortality and cancer-specific mortality [18].

The key independent variables of the current study were the interaction effects of individual SES and neighborhood SES on survival. Survival of each NPC patient was determined by linking their 2002 to 2006 mortality data with claims data for first curative treatment up to five years prior to death. With these data, we could calculate death-free survival. Patient characteristics included age, gender, geographic location, treatment modality, severity of disease, and monthly income. Disease severity was based on the modified Charlson Comorbidity Index Score (CCIS), which is widely accepted for risk adjustment in administrative claims data sets [19].

### Individual-level Measures

The four-factor Hollingshead scale uses marital status, gender, education and occupation [20]. In this series, we used enrollee category (EC) as a proxy of individual socioeconomic status which had been validated in previous studies [11]. Enrollee category, which defines workplace, is an important prognostic factor for cancer [21],[22]. In Taiwan, the NHRID classified people into four subgroups: EC 1 (civil servants, full-time, or regularly paid personnel with a government affiliation), EC 2 (employees of privately owned institutions), EC 3 (self-employed individuals, other employees, and members of the farmers’ or fishermen’s association), EC 4 (veterans, members of low-income families, and substitute service draftees). According to EC, the NPC patients in our study were then further classified into three subgroups: EC 1–2 (high SES), EC 3 (moderate SES), EC 4 (low SES).

### Neighborhood-level Socioeconomic Status

Neighborhood SES is a contextual factor based on neighborhood household income averages and percentages reported in Taiwan’s 2001 Census. In that census, neighborhood household income was measured by township using per capita income which could be determined based on 2001 tax statistics released by Taiwan’s Ministry of Finance, (http://www.fdc.gov.tw/dp.asp?mp=5). The categorization into advantaged or disadvantaged neighborhoods was based on the median values, with advantaged neighborhoods having higher-than-median neighborhood household incomes and disadvantaged neighborhoods having lower-than-median household incomes.

### Other Variables

We used population density, percentage of residents with college level or higher education, percentage of residents >65 years old, percentage of residents who were agriculture workers, and the number of physicians per 100000 people to categorize urbanization level of residences into one of seven levels [23]. The urban level was categorized as level 1, suburban level was subcategorized into levels 2 and 3, and rural level was subcategorized into levels 4 to 7.

The hospitals were categorized by hospital teaching level (medical center, regional hospital, or district hospital). The geographic regions were recorded as northern, central, southern and eastern Taiwan.

### Statistical Analysis

All statistical operations were performed using SPSS (version 15, SPSS Inc., Chicago, IL, USA). Pearson’s chi-square test was used for categorical variables such as gender, level of urbanization, geographic regions of residence, category of Charlson Comorbidity Index Score, treatment modality, tumor extent, and hospital characteristics (teaching level, ownership, and caseload). Continuous variables were analyzed by one-way ANOVA.

The cumulative 5-year survival rates and the survival curves were constructed and compared using the log-rank test. Survival curves, which were stratified by individual SES and neighborhood SES, were measured from the time of diagnosis by using overall mortality as the event variable.

The Cox proportional hazards regression model was used to compare outcomes of different SES categories after adjusting for patients’ characteristics (age, gender, Charlson Comorbidity Index Score, urbanization and area of residence), treatment modality (radiotherapy, chemotherapy, chemoradiotherapy) and hospital characteristics. Low individual SES and disadvantaged neighborhood group as the reference group. A two-sided *p*-value (*p*<0.05) was considered significant.

In order to explore whether the impact of combined individual and neighborhood SES on NPC survival rates is robust, we further adopted the insurance income as a proxy of individual SES. After merging with the neighborhood SES, the analysis was performed as the above mentioned procedures.

## Results

### Demographic Data and Clinical Characteristics

A total of 4691 NPC patients who had received treatment were included in this study (Table 1). The mean age at diagnosis differed significantly by individual SES. In the high individual SES group, mean age at diagnosis was 45 years old; in the moderate individual SES group, it was 52, and in the low individual SES group 54 (*P*<0.001). Because we found interaction effects between age and several other variables, we further stratified the patients into two groups–those below 65 years old and those 65 years old and older.

**Table 1:** Baseline characteristics (n = 4691).

Variables	Age <65 years (n = 4001)							Age ≧65 years (n = 690)						
	High SES		Moderate SES		Low SES		p value	High SES		Moderate SES		Low SES		p value
	(n = 1685)		(n = 1453)		(n = 863)			(n = 72)		(n = 282)		(n = 336)		
Mean age, years (±SD)	44	±9.4	49	±8.9	47	±11.4	<0.001	70	±4.4	72	±5.3	73	±5.6	0.002
Gender							<0.001							0.035
Male (%)	1335	(79.2)	1051	(72.3)	594	(68.8)		57	(79.2)	230	(81.6)	245	(72.9)	
Female (%)	350	(20.8)	402	(27.7)	269	(31.2)		15	(20.8)	52	(18.4)	91	(27.1)	
Urbanization							<0.001							<0.001
Urban (%)	622	(36.9)	331	(22.8)	232	(26.9)		22	(30.6)	8	(2.8)	104	(31.0)	
Suburban (%)	791	(46.9)	662	(45.6)	390	(45.2)		39	(54.2)	86	(30.5)	162	(48.2)	
Rural (%)	272	(16.1)	460	(31.7)	241	(27.9)		11	(15.3)	188	(66.7)	70	(20.8)	
Geographic Region							<0.001							<0.001
Northern (%)	964	(57.2)	589	(40.5)	398	(46.1)		43	(59.7)	82	(29.1)	184	(54.8)	
Central (%)	256	(15.2)	240	(16.5)	168	(19.5)		10	(13.9)	62	(22.0)	46	(13.7)	
Southern/Eastern (%)	465	(27.6)	624	(42.9)	297	(34.4)		19	(26.4)	138	(48.9)	106	(31.5)	
Charlson Comorbidity Index Score							0.072							0.171
0 (%)	612	(36.3)	499	(34.3)	305	(35.3)		21	(29.2)	87	(30.9)	95	(28.3)	
1–6 (%)	810	(48.1)	691	(47.6)	387	(44.8)		35	(48.6)	145	(51.4)	197	(58.6)	
>6 (%)	263	(15.6)	263	(18.1)	171	(19.8)		16	(22.2)	50	(17.7)	44	(13.1)	
Treatment modality							0.126							0.012
Radiotherapy (%)	184	(10.9)	187	(12.9)	111	(12.9)		15	(20.8)	72	(25.5)	120	(35.7)	
Chemotherapy (%)	213	(12.6)	180	(12.4)	128	(14.8)		11	(15.3)	37	(13.1)	50	(14.9)	
Chemoradiotherapy (%)	1288	(76.4)	1086	(74.7)	624	(72.3)		46	(63.9)	173	(61.3)	166	(49.4)	
Hospital characteristics							0.001							
Teaching level														0.083
Medical center (%)	1173	(69.6)	1034	(71.2)	588	(68.1)		41	(56.9)	172	(61.0)	233	(69.3)	
Regional (%)	500	(29.7)	385	(26.5)	256	(29.7)		28	(38.9)	91	(32.3)	89	(26.5)	
District (%)	12	(0.7)	34	(2.3)	19	(2.2)		3	(4.2)	19	(6.7)	14	(4.2)	

Abbreviation: SES, socioeconomic status.

NPC patients below the age of 65 years old with moderate and low individual SES were more likely to be older, to reside in rural areas, especially in southern and eastern Taiwan, and to receive treatment in district hospitals than their high individual SES counterparts (*P*≦0.001, respectively).

Patients aged 65 and above with moderate and low individual SES were more likely to be older, to reside in rural area, especially in southern and eastern Taiwan, and to undergo radiotherapy alone than those with high individual SES in the same age group (*P* = 0.002, <0.001 and 0.012, respectively). No significant difference was found in treatment hospital characteristics between the SES groups (*P* = 0.083).

### Univariate Survival Analysis

**Figure 1:**
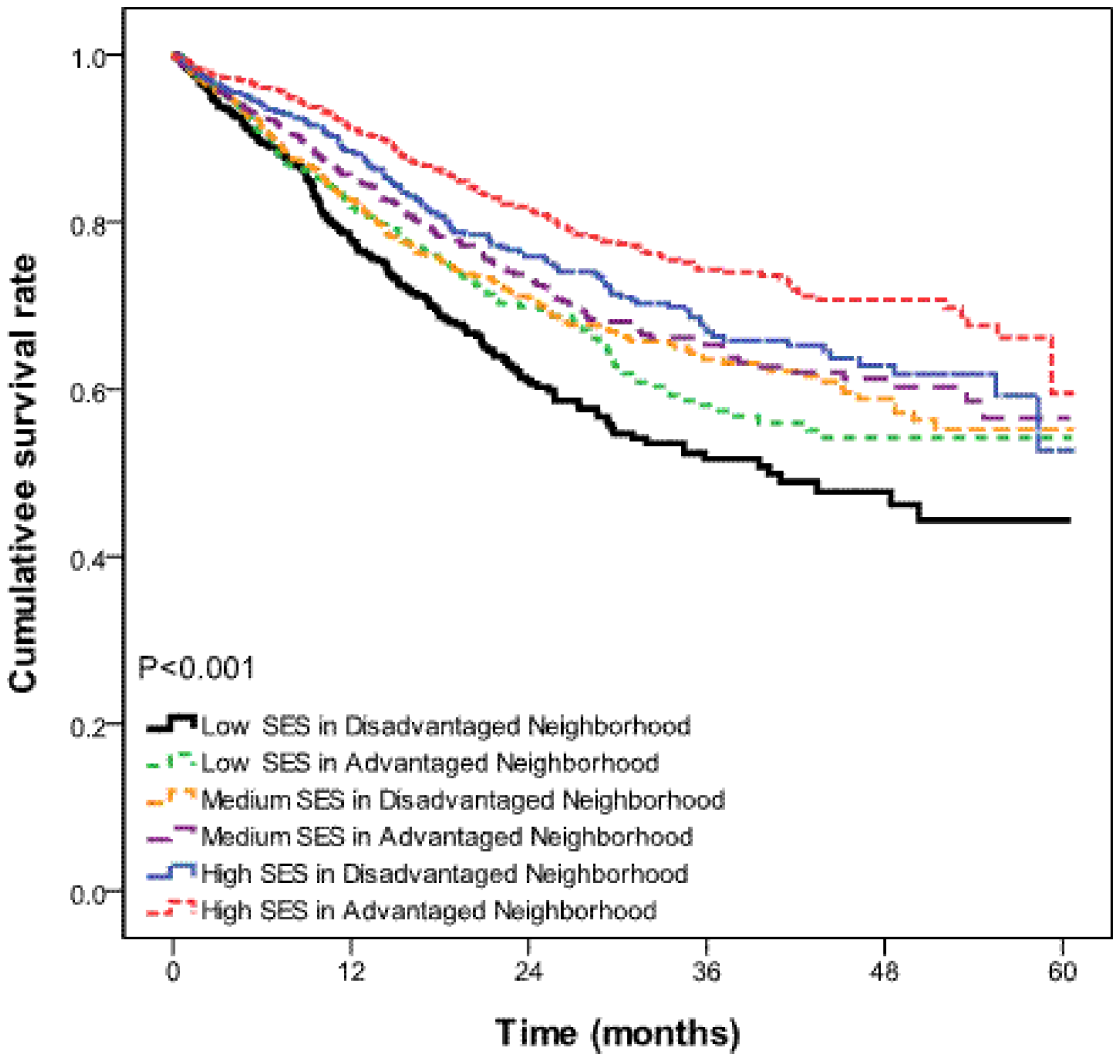
Survival curves by individual-level and neighborhood-level SES for NPC patients aged below 65 years.

**Figure 2:**
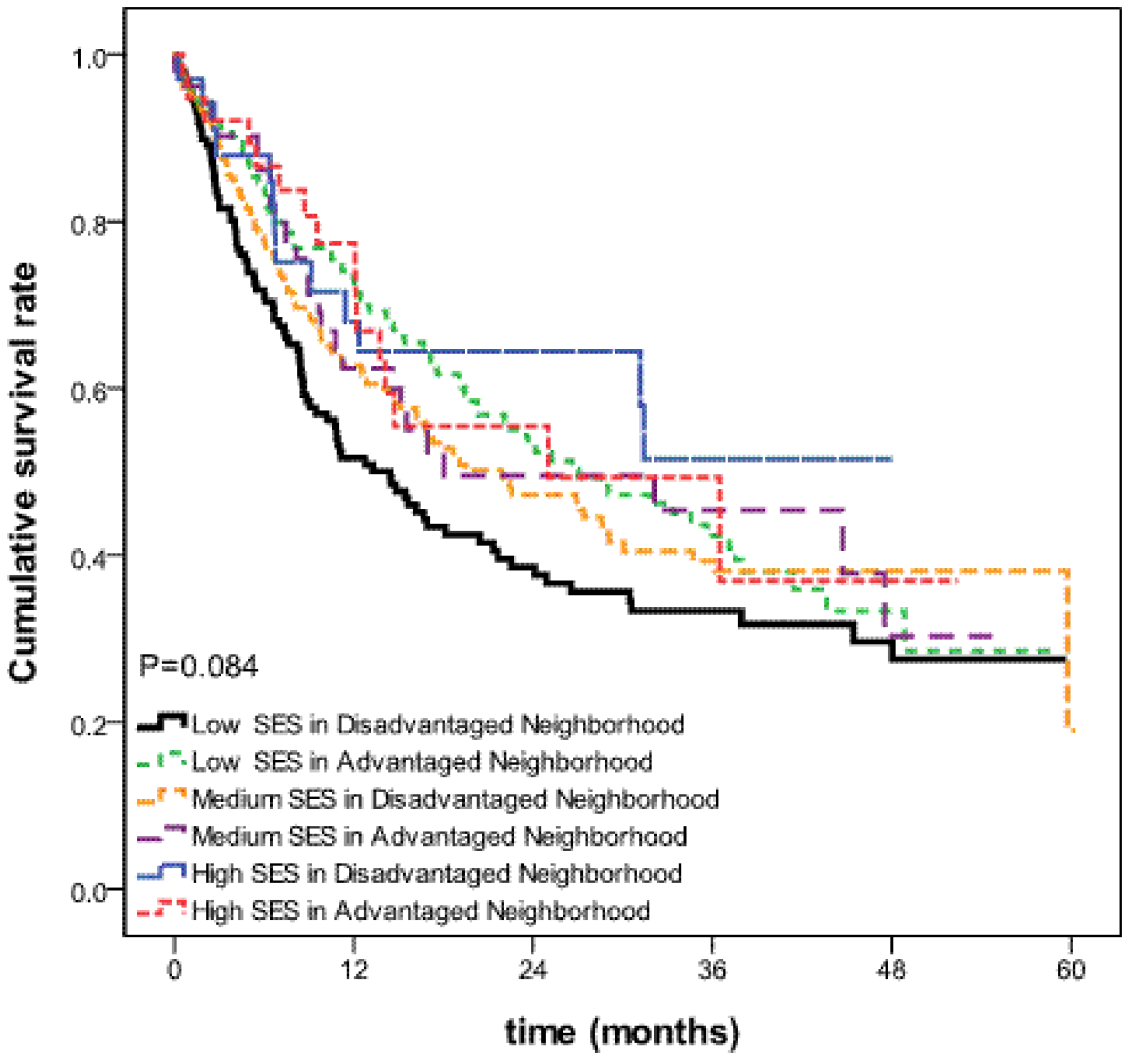
Survival curves by individual-level and neighborhood-level SES for NPC patients aged 65 years and above.

As can be seen in Table 2, among the NPC patients below 65 years old, those categorized as low SES residing in disadvantaged neighborhoods had significantly worse survival rate than all comparison groups (all, p<0.001). For those 65 years old and above, we found no significant difference between SES and 5-year survival rates (Table 2). These results are depicted graphically in Figures 1 and 2.

**Table 2:** Combined effect of individual SES and neighborhood SES on 5-year overall survival rates in nasopharyngeal cancer patients (n = 4691).

	Neighborhood socioeconomic status	Individual socioeconomic status															
		Age <65 years (n = 4001)								Age ≧65 yeasr (n = 690)							
		Low SES		Moderate SES		High SES		p value		Low SES		Moderate SES		High SES			p value
Nasopharyngeal cancer (n = 4691)		Total no.	Alive no. (%)	Total no.	Alive no. (%)	Total no.	Alive no. (%)	<0.001	Total no.		Alive no. (%)	Total no.	Alive no. (%)		Total no.	Alive no. (%)	0.084
	Disadvantaged	441	274 (62.1)	763	545 (71.4)	675	517 (76.6)		149		58 (38.9)	229	114 (49.8)		34	21 (61.8)	
	Advantaged	422	285 (67.5)	690	507 (73.5)	1010	820 (81.2)		187		100 (53.5)	53	27 (50.9)		38	22 (57.9)	

Abbreviation: SES, socioeconomic status.

### Multivariable Survival Analysis

The result of our univariate survival analysis indicated the presence of interaction effects between patient age and survival rates by SES. In the Cox proportional hazards regression model we used for our multivariate analysis, the combined effect of individual SES and neighborhood SES remained significant after adjusting for other factors in patients under 65 years old. Adjusted hazard ratios revealed that, in this age group, those with moderate or high individual SES had a 0.5–0.73-fold lower risk of death than those the same age with low individual SES residing in disadvantaged neighborhoods (Table 3). No significant differences in these relationships were found among those 65 years old and above.

**Table 3:** Hazard ratios of individual SES for mortality in advantaged and disadvantaged neighborhoods*.

	Neighborhood socioeconomic status	Individual socioeconomic status											
		Age <65 years (n = 4001)						Age ≧65 years (n = 690)					
		Low SES		Moderate SES		High SES		Low SES		Moderate SES		High SES	
		Adjusted HR	95% CI	Adjusted HR	95% CI	Adjusted HR	95% CI	Adjusted HR	95% CI	Adjusted HR	95% CI	Adjusted HR	95% CI
Nasopharyngeal cancer(n = 4691)		n = 4001						n = 690					
	Disadvantaged	1		0.77	0.63–0.94	0.72	0.58–0.90	1		0.81	0.60–1.11	0.71	0.39–1.30
	Advantaged	0.91	0.72–1.16	0.71	0.56–0.88	0.54	0.43–0.67	0.88	0.64–1.22	0.87	0.56–1.36	0.79	0.46–1.37

Abbreviation: Adjusted HR, adjusted hazard ratio; 95% CI, 95% confidence interval; SES, socioeconomic status.

* Adjusted for the patients’ diagnosed age, gender, Charlson Comorbidity Index Score, urbanization and region of residence, adjuvant therapy, and hospital characteristics.

Table 4 shows that NPC patients with low SES in disadvantaged neighborhood had lower healthcare resources, such as physicians per 10000 residents and pharmacists in both younger and older groups. High-SES patients in advantaged neighborhood were more likely to have higher level of education and higher median household income. These data supported the reason why we used six individual and neighborhood SES groups.

**Table 4:** Sociodemographic characteristics by individual and neighborhood socioeconomic status.

	High individual SES		Moderate individual SES		Low individual SES		p value
	Advantaged neighborhood	Disadvantaged neighborhood	Advantaged neighborhood	Disadvantaged neighborhood	Advantaged neighborhood	Disadvantaged neighborhood	
**Age <65 years**							
Number of patients	1010	675	690	763	422	411	
Number of deaths (%)	190 (18.8)	158 (23.4)	183 (26.5)	218 (28.6)	137 (32.5)	167 (37.9)	<0.001*
Mean age, mean±SD	43.86±9.46	44.32±9.35	48.39±8.73	48.62±9.12	47.92±11.66	46.04±11.05	<0.001
Male gender, %	798 (79)	537 (79.6)	511 (74.1)	540 (70.8)	285 (67.5)	309 (70.1)	<0.001*
Education≧high school, %	98.6%	84.4%	99.1%	61.9%	98.8%	66.7%	<0.001*
Median household income, NT$1000	624±70	497.07±33.46	596±55	480±38	607±59	485±42	<0.001
Health care-related resources							
Physicians per 10,000 persons, mean±SD	26±21	12±10	25±20	12±10	24±20	10±9	<0.001
Pharmacists per 10,000 persons, mean±SD	10±11	4±3	7±4	4±3	9±9	4±3	<0.001
**Age ≧65 years**							
Number of patients	38	34	53	229	187	149	
Number of deaths (%)	16 (42.1)	13 (38.2)	26 (49.1)	115 (50.2)	87 (46.5)	91 (61.1)	<0.001*
Mean age, mean±SD	70.32±4.59	69.87±4.34	71.91±5.73	72.19±5.23	72.08±5.71	73.29±5.40	0.004
Male gender, %	27 (71.1)	30 (88.2)	47 (88.7)	183 (79.9)	142 (75.9)	103 (69.1)	0.017*
Education≧high school, %	100%	88.2%	98.1%	43.2%	97.3%	75.2%	<0.001*
Median household income, NT$1000	620±63	501±32	591±42	472±36	614±66	490±40	<0.001
Health care-related resources							
Physicians per 10,000 persons, mean±SD	30±22	10±6	18±11	9±10	27±23	12±12	<0.001
Pharmacists per 10,000 persons, mean±SD	7±5	4±2	5±3	3±2	8±8	4±3	<0.001

* Pearson’s chi-square test.

We further used insurance income as a proxy of individual SES and then merged with the neighborhood SES. The results were similar to the above mentioned results (Appendix S1). After adjusting other factors, the combined effect of individual SES and neighborhood SES remained more significant in patients under 65 years old (Appendix S2).

## Discussion

This study found that, among NPC patients aged below 65 years old in Taiwan, those with low individual SES residing in disadvantaged neighborhoods were at 2-fold higher risk of mortality than those with high SES living in advantaged neighborhoods after adjusting age at diagnosis, gender, and CCIS. No such significant differences were found in those 65 years old and above. To the best of our knowledge, this work is the first to evaluate the combined effect of individual and neighborhood SES on the risk of NPC mortality in a population-based study using data provided by a national health insurance system.

While SES has been shown to significantly impact survival in head and neck cancer [6], its role in NPC survival has rarely received such research attention. There have been a few reports focusing on SES and NPC incidence [24], [25]. Turkoz et al., for example, observed significant association between low SES and elevated NPC risk. Munck et al. found that patients being treated for Waldeyer’s ring carcinoma (including NPC, tonsil cancer, and tongue base cancer) in a public hospital setting had significant delayed treatment compared with patients at an academic center [26]. However, neither of these reports investigated whether SES or the social environment might contribute to the prognosis of NPC.

Neighborhood features that may affect prognosis of NPC can be classified into characteristics of the physical environment and characteristics of the social environment. Whether or not one lives in a disadvantaged community may contribute to inequality of medical resource or higher frequency of deleterious behaviors that influence the survival rate of NPC. Among NPC patients under 65 years old in our study, those with low SES living in disadvantaged neighborhoods had the highest risk of mortality. These patients tended to live in rural areas, live in southern and eastern Taiwan, and undergo treatment in regional or district hospitals, which would suggest that the existence of an inequality in available hospital resources such as available diagnostic tools and available modalities of treatment modalities. MRI imaging has now virtually replaced CT scans as a means of staging tumors, including NPC prior to treatment [27]. However, in Taiwan, it may be too expensive for a regional or district hospital to afford a high-resolution MRI machine. Kam et al. found that intensity-modulated radiotherapy (IMRT), another very expensive piece of equipment, offers better tumor coverage and normal organ sparing in locally advanced NPC and allows more room for dose escalation than three-dimensional conformal radiotherapy (3-D CRT) [28]. Therefore, hospitals without this imaging modality may not be able to offer their patients these treatment advantages. In addition, where patients reside and the level of hospital they visit may also affect physician caseload, a possible proxy for experience. Lee et al. showed that NPC patients who were treated by high caseload volume physicians had a lower risk of death and were more likely to have greater survival [29]. Academic medical centers, which have more resources and a greater number of cases, are more likely to offer high-resolution MRI or positron emission tomography (PET) scan for tumor staging, IMRT with or without chemotherapy for radiotherapy and have physicians with high caseload volumes. Since in Taiwan these centers are most often located in urban areas and advantaged neighborhoods, NPC patients residing in these locations are more likely able to enjoy improvement mortality rates.

Part of the effect of neighborhood can also be related to social norms and prevailing attitudes toward health and health-related behavior (e.g., smoking, salted fish) and features of the social connections within neighborhoods such as social cohesion and support. Theoretically, NPC patients with low SES living in disadvantaged neighborhood may receive less to social support, be at more risk of negative affect/depression, have more stress and less social capital, and have less access to positive collective social influence [30], [31]. Symptoms related to NPC in the early stage are usually nonspecific until tumor spreading to lymph nodes in the neck. These factors may contribute to diagnosis at later stages and lower access to quality treatment, and aggravate the risk of death among patients with low SES residing in disadvantaged neighborhoods. An informed circle of family and friends would more likely encourage such patients to seek medical attention earlier than they would on their own.

Some cancers, including colorectal cancer and breast cancer, have been found that disparities in treatment and SES are associated with decreased survival rate [32], [33]. Physician treatment decisions may affect the outcomes of NPC patients with low SES. Concurrent cisplatin-based chemoradiotherapy has been demonstrated to provide significant survival improvement and is currently the standard treatment strategy for NPC patients with locoregional advanced disease [34]. Nevertheless, concurrent chemoradiotherapy is associated with significant comorbidity, including severe nutrient deficiency, leucopenia, nephrotoxicity, transverse myelitis and central nervous system disease [35], [36]. In order to avoid these side effects and improve the treatment compliance, physicians may not prescribe chemotherapy for NPC patients with low SES living in disadvantaged neighborhoods because they may assume their patients can not adhere to prescribed treatment. In contrast, patients with high SES living in advantaged neighborhood have better access to modern facilities with more resources than those living in disadvantage neighborhoods and may be better able to afford medicine at their own expense, such as helical tomotherapy, image-guided radiation therapy, radioprotective or effective antiemetic agents. This would allow them to complete the evidence-based therapies and reduce treatment toxicity. Thus, physicians would be more likely to prescribe such treatment modalities to high SES patients living in advantage neighborhoods.

Our series did not find a significant relationship between SES and survival rate in NPC patients aged 65 years old and above. Similarly, Chang et al. did not find significant relationships between these two variables in lung cancer, breast cancer, colorectal cancer and head and neck cancer in Taiwan [37]. The reason for the reduced significance of this relationship in this age group may be related to their increased competing mortality and poor tolerance for high doses of radiation. A study by Kim and Durden [38] reported that the relationship between income and physical impairment also diverged with increasing age. Therefore, the relevance of SES at the neighborhood and individual level may lose significance because eventually with increasing age people have more health problems (e.g., diabetes, hypertension, stroke, or myocardial infarction) and become frailer, which would make them less tolerant of radiotherapy. Therefore, it is no surprise that one clinical study of NPC in Asia found the 5-year survival rate for the youngest age group (<70 years) to be 78.1% and the eldest age group (>70 years) to be 43.9% [39].

The findings of this large population-based study assessing of combined effect individual SES and neighborhood SES on mortality in NPC patients underscore need for better treatment information, access to modern diagnostic and therapeutic modalities, quality of therapy, service availability, and social support for NPC patients residing in disadvantaged neighborhoods. Physicians treating NPC patients should be aware that SES has on clinical outcomes and that some of their patients, especially those with low SES residing in disadvantage neighborhoods, may face additional challenges to their survival rate.

This study has several limitations. One limitation is that the diagnosis of NPC as well as any other comorbidity in this study was garnered from ICD codes on National Health Insurance claims. While this is not ideal, the National Health Insurance Bureau in Taiwan does randomly review the charts and interviewed the patients to spot verify the accuracy of diagnosis. Another limitation was our lack of access to detailed information from the insurance claims database with regard to NPC stage, pattern of relapse, and other risk factors that may impact NPC survival, such as tobacco, salted fish feeding and Epstein-Barr virus status [40],[41]. These may be the important variables for increased mortality rate among NPC patients with low individual SES residing in disadvantaged neighborhood. Delayed diagnosis or lack of access to screen in low SES patients would lead to more advanced cancer stage at diagnosis and reduce possibility of treatment after diagnosis. Besides, SES is generally related to quality of life, nourishment level and dietary habits which may affect NPC survival. For example, Fang et al. reported that lower SES was significantly correlated with worse health-related quality of life among treated NPC survivors [42]. An individual patient’s inferior capabilities and resources would hinder them from coping with cancer threats and complications. Low quality of life and bad nourishment might increase the risk of mortality among NPC patients. Therefore, further study should be designed to compare the effect of different variables on NPC mortality rate by using quality of life instrument and cancer registry data with more information. Still another limitation is the lack specific data regarding treatment techniques, such as chemotherapy regimen, radiation dose, and they type of radiotherapy instruments used. This prevented further subgroup analysis. However, given the robustness of the evidence and statistical analysis in this study, these limitations are unlikely to compromise our results.

In conclusion, this study is the first to link the combined effect of individual SES and neighborhood SES to 5-year overall survival of NPC. The finding of high risk of mortality among NPC patients with low individual SES who live in disadvantaged neighborhood suggests public health efforts may need to address existing socioeconomic disparities in health in NPC patients.

## Supporting Information

Appendix S1 The combined effect of individual and neighborhood SES on NPC survival rates in patients aged less than 65 years (a) and those aged 65 years and above (b).
(DOC)

Appendix S2 Hazard ratios of individual SES (defined by insurance income) for mortality in advantaged and disadvantaged neighborhoods.
(DOC)
