# Supplementary material for: The Combined Effect of Individual and Neighborhood Socioeconomic Status on Nasopharyngeal Cancer Survival
Source: PLoS One. 2013 Sep 12;8(9):e73889. doi: 10.1371/journal.pone.0073889 (PMC3771923; doi:10.1371/journal.pone.0073889)
Supplement: Appendix S1 — The combined effect of individual and neighborhood SES on NPC survival rates in patients aged less than 65 years (a) and those aged 65 years and above (b). (DOC) [file pone.0073889.s001.doc]

**Appendix S1**

**(a)**

**(b)**

**The combined effect of individual (defined by insurance income) and neighborhood SES on NPC survival rates in in patients aged less than 65 years (a) and those aged 65 years and above (b).**
