# Supplementary material for: The Combined Effect of Individual and Neighborhood Socioeconomic Status on Nasopharyngeal Cancer Survival
Source: PLoS One. 2013 Sep 12;8(9):e73889. doi: 10.1371/journal.pone.0073889 (PMC3771923; doi:10.1371/journal.pone.0073889)
Supplement: Appendix S2 — Hazard ratios of individual SES (defined by insurance income) for mortality in advantaged and disadvantaged neighborhoods. (DOC) [file pone.0073889.s002.doc]

| **Appendix S2** Hazard ratios of individual SES (defined by insurance income) for mortality in advantaged and disadvantaged neighborhoods* | | | | | | | | | | | | | |
| --- | --- | --- | --- | --- | --- | --- | --- | --- | --- | --- | --- | --- | --- |
|  | Neighborhood  socioeconomic  status | Individual socioeconomic status | | | | | | | | | | | |
|  |  | Age <65 years (n=4001) | | | | | | Age ≧65 years (n=690) | | | | | |
|  |  | Low SES | | Moderate SES | | High SES | | Low SES | | Moderate SES | | High SES | |
|  |  | Adjusted  HR | 95%  CI | Adjusted  HR | 95% of CI | Adjusted  HR | 95%  CI | Adjusted  HR | 95%  CI | Adjusted  HR | 95%  CI | Adjusted  HR | 95%  CI |
| Nasopharyngeal cancer(n=4691) | | n=4001 | | | | | | n=690 | | | | | |
|  | Disadvantaged | 1 |  | 0.27 | 0.21-0.35 | 0.42 | 0.31-0.56 | 1 |  | 0.10 | 0.05-0.22 | 0.19 | 0.03-1.38 |
|  | Advantaged | 0.80 | 0.68-0.95 | 0.39 | 0.31-0.48 | 0.32 | 0.25-0.41 | 0.78 | 0.60-1.01 | 0.41 | 0.15-1.12 | 1.04 | 0.38-2.86 |
| Abbreviation: Adjusted HR, adjusted hazard ratio; 95% CI, 95% confidence interval; SES, socioeconomic status.  * Adjusted for the patients’ gender, age, urbanization, geographic region, Charlson Comorbidity Index Score, treatment modality, and hospital characteristics. | | | | | | | | | | | | | |
